# Supplementary material for: Environmental dynamics impact whether matching is optimal
Source: PNAS Nexus. 2025 Dec 17;5(1):pgaf392. doi: 10.1093/pnasnexus/pgaf392 (PMC12767605; doi:10.1093/pnasnexus/pgaf392)
Supplement: pgaf392_Supplementary_Data [file pgaf392_supplementary_data.pdf]

Supplementary Information:
Environmental dynamics impact whether matching is optimal

Yipei Guo[1,2†] and Ann M. Hermundstad[1†]

[1]Janelia Research Campus, Howard Hughes Medical Institute, Ashburn, VA, USA
[2]Institute of High Performance Computing, Agency for Science, Technology and Research, Singapore
[†] for correspondence: guo_yipei@a-star.edu.sg, hermundstada@janelia.hhmi.org

1 Optimality condition

For a given fixed sampling rate policy $\vec{p} = [p_1, p_2, ..., p_N]$ with $\sum_i p_i \leq 1$, the overall average collection rate is given by: $\langle c \rangle = c_1 + c_2 + ... + c_N$, where $c_i(p_i) = p_i P_{c,i}(p_i)$ is the collection rate at an option i and $P_{c,i}(p_i)$ is the corresponding collection probability. Solving for the optimal policy $\vec{p}^*$ then corresponds to finding the sampling rates that maximizes $\langle c \rangle$ subject to the constraints that $\sum_i p_i \leq 1$ and $p_i \geq 0$ for all i.

For all the replenishment structures that we consider in this paper, $\left.\frac{dc_i}{dp_i}\right|_{p_i=0}$ takes on the same value (of 1), and $c_i(p_i)$ exhibits diminishing returns, i.e., $\frac{dc_i}{dp_i}$ decreases with p_i. This implies that if an agent is not sampling one of the options at all (e.g. $p_j = 0$ for option j) and sampling another option at a finite rate (e.g. $p_k > 0$ for option k), it is always possible to improve the overall average collection rate by decreasing the sampling rate at option k by a small amount δp and instead sample option j at rate δp (such that the total sampling rate is fixed). Formally, this is because the change in the average collection rate due to such a perturbation is given by: $\delta\langle c \rangle = \delta c_j + \delta c_k = \left(\frac{dc_j}{dp_j} - \frac{dc_k}{dp_k}\right)\delta p > 0$. This implies that the optimal sampling rate at each of the options must be strictly positive ($p_i^* > 0 \, \forall \, i$). Therefore, the only potentially active constraint in our optimization problem is the one that involves the total sampling rate.

The optimal policy is therefore one that minimizes the following Lagrangian function:

$$L(p_1, p_2, ..., p_N, \lambda) = -\sum_i c_i(p_i) + \lambda \left(\sum_i p_i - 1 + q^2 \right), \tag{S1}$$

where we have introduced the slack variable q for the constraint.

The corresponding gradients of L with respect to its variables are then given by:

$$\frac{\partial L}{\partial p_i} = -\frac{\partial c_i}{\partial p_i} + \lambda \tag{S2}$$

$$\frac{\partial L}{\partial \lambda} = \sum_i p_i - 1 + q^2 \tag{S3}$$

If the constraint is active ($q = 0$), the agent samples at the maximum possible rate ($\sum_i p_i = 1$ such that $\frac{\partial L}{\partial \lambda} = 0$), and the policy is optimal if:

$$\frac{\partial r_i}{\partial p_i} = \lambda \quad \text{for all options } i. \tag{S4}$$

If the constraint is inactive ($\lambda = 0$ and $q^2 > 0$), the agent samples less frequently that the maximum possible rate ($\sum_i p_i < 1$), and the policy is optimal if:

$$\frac{\partial c_i}{\partial p_i} = 0 \quad \text{for all options } i. \tag{S5}$$

Therefore, in any given environment, the optimal policy $\vec{p}^*$ is one where $\left.\frac{\partial c_i}{\partial p_i}\right|_{p_i^*}$ is the same across all options.

It is useful to note that when $c_i(p_i)$ is a monotonically increasing function for at least one of the options in the environment, which is always the case for replenishment structures (A) and (B) regardless of the replenishment statistics, it is beneficial to sample as fast as possible and hence the constraint will be active ($q = 0$). In other words, in the space of replenishment processes we consider, an optimal policy with $\sum_i p_i < 1$ is only possible if all options have replenishment structure of type (C).

1.1 Optimality condition in the presence of replenishment rate fluctuations

If the replenishment rate θ_i of an option i is fixed, $P_c(p_i|\theta_i)$ is the collection probability given a sampling rate p_i at that option. In the presence of replenishment rate fluctuations (in the form of changes in replenishment rates at regular intervals), if the values of θ_i are drawn from a distribution $P(\theta_i)$, the corresponding average collection probability is then given by:

$$\langle P_c(p_i)\rangle = \int P_c(p_i|\theta_i)P(\theta_i)d\theta_i \tag{S6}$$

and the optimal policy is the one that maximizes the average collection rate $\langle c\rangle = \langle c_1\rangle + \langle c_2\rangle + ... + \langle c_N\rangle$, with $\langle c_i(p_i)\rangle = p_i\langle P_c(p_i)\rangle$.

Repeating the derivation for the optimality condition while taking into account the averaging over the distributions of replenishment rates, we find that the optimal policy $\vec{p}^*$ is one where $\left.\frac{\partial\langle c_i\rangle}{\partial p_i}\right|_{p_i^*} = \left.\left\langle \frac{\partial c_i}{\partial p_i}\right\rangle\right|_{p_i^*}$ is the same across all options.

2 Relationship between the average marginal gain $\langle g\rangle$ and the average collection probability $\langle P_c\rangle$ in the presence of fluctuations or uncertainty

2.1 For a given replenishment structure and statistics, the relationship between $\langle g\rangle$ and $\langle P_c\rangle$ depends only on the coefficient of variation CV of $P(\theta)$.

We will assume that the distribution of replenishment rate $P(\theta)$ is a 2-parameter distribution, with both parameters jointly determining the mean $\bar{\theta}$ and standard deviation σ_θ of $P(\theta)$. The coefficient of variation is then defined to be $CV_\theta = \sigma_\theta/\bar{\theta}$. It is useful to define the scaled replenishment rate $\tilde{\theta} = \theta/\bar{\theta}$, such that $P(\tilde{\theta})$ has mean $\langle\tilde{\theta}\rangle = 1$ and standard deviation $\sigma_{\tilde{\theta}} = CV_\theta$.

For any function $f(p/\theta)$ that depends only on the ratio between the sampling rate and the replenishment rate, the average of the function over $P(\theta)$ is given by:

$$\begin{aligned}\langle f\rangle &= \int f\left(\frac{p}{\theta}\right)P(\theta)d\theta \\ &= \int f\left(\frac{\tilde{p}}{\tilde{\theta}}\right)P(\tilde{\theta})d\tilde{\theta},\end{aligned} \tag{S7}$$

where $\tilde{p} = p/\bar{\theta}$.

Since $P(\tilde{\theta})$ is a 1-parameter distribution with that parameter determined by CV_θ, $\langle f\rangle$ is a function of both $\tilde{p}$ and CV_θ. We saw in the main text that both the marginal gain g (Eqs. 5, 8, 11 in the main text) and the collection probability P_c (Eqs. 4, 7, 10 in the main text) are just functions of $\tilde{p}$. Therefore, if all options in the environment have the same replenishment structure and statistics but differ in their mean replenishment rates, even when their replenishment rates are not fixed, as long as they have the same degree of fluctuation CV_θ, the relationship between $\langle g\rangle$ and $\langle P_c\rangle$ is the same for all options, in which case the optimal policy gives rise to matching.

2.2 Increasing the degree of fluctuation CV for an option reduces the ratio of its collection probability to that of the other options under the optimal policy.

We analyzed the relationship between $\langle g\rangle$ and $\langle P_c\rangle$ for different replenishment structures and statistics, and for different distributions of replenishment rates $P(\theta)$. We considered both the uniform distribution and the gamma distribution for $P(\theta)$ (Fig. S1A) and found that in both cases, increasing CV of the distribution reduces $\langle P_c\rangle$ for any given $\langle g\rangle$ that lies between 0 and 1 (Fig. S1B,C), This is the case across all the different environments and implies that under the optimal policy, $\langle P_c\rangle$ is lower for options with a higher degree of fluctuation.

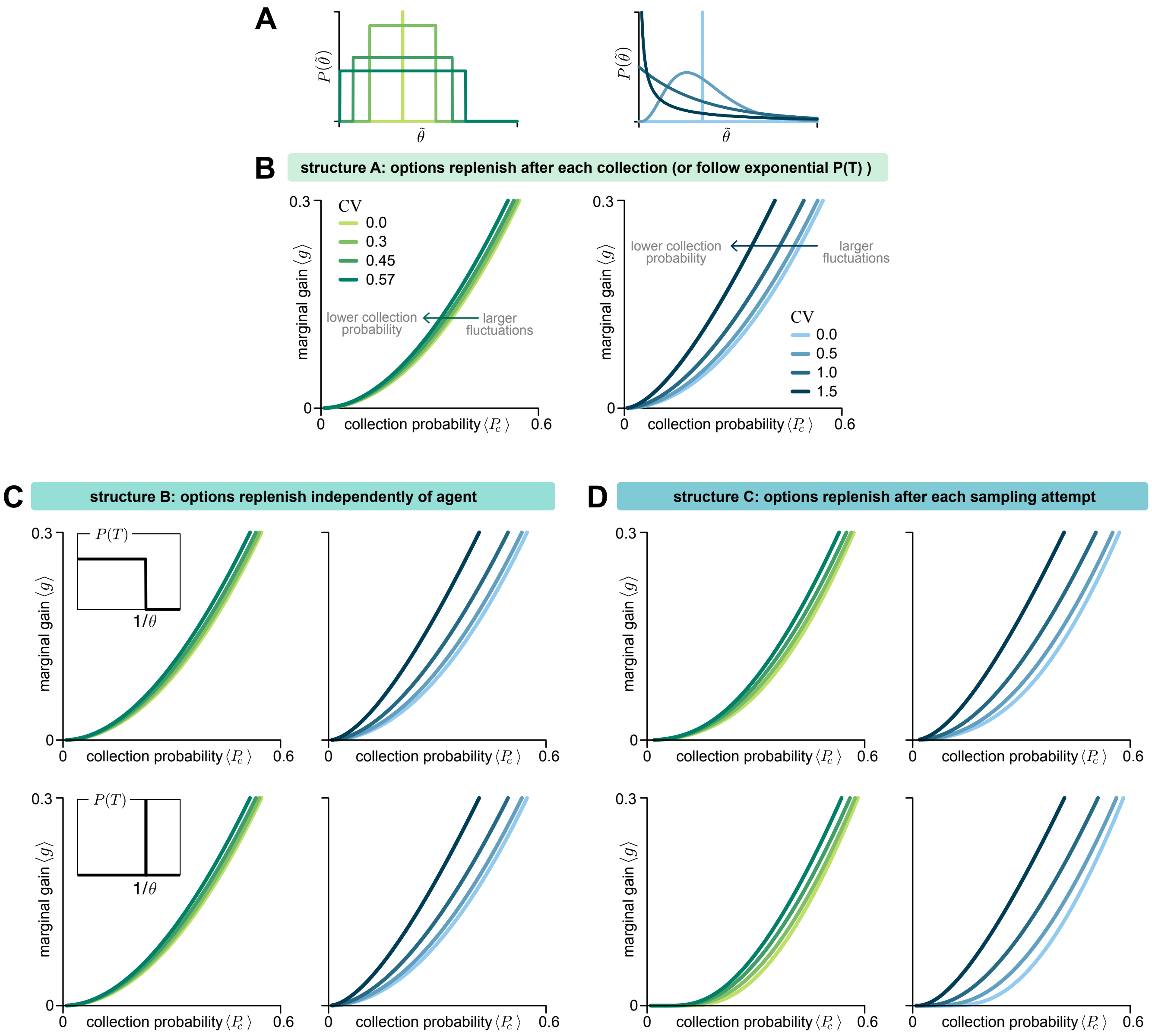

Figure S1: **The impact of fluctuations on the relationship between marginal gain and collection probability is consistent across different environments. A)** We considered uniform distributions (left) and gamma distributions (right) for the distribution of replenishment rates $P(\theta)$. **B)** The relationship between $\langle g \rangle$ and $\langle P_c \rangle$ when the replenishment process is of structure type (A), or if $P(T)$ is exponential. **C-D)** The relationship between $\langle g \rangle$ and $\langle P_c \rangle$ for other replenishment structures and statistics (top row: $P(T)$ is uniform; bottom row: $P(T)$ is a delta-distribution.)
